# A Low-Cost Efficient Multiplex PCR for Prenatal Sex Determination in Bovine Fetus Using Free Fetal DNA in Maternal Plasma

**Published:** 2012-06-19

**Authors:** Arash Davoudi, Ramin Seighalani, Seyed Ahmad Aleyasin, Alireza Tarang, Abdolreza Salehi Salehi, Farideh Tahmoressi

**Affiliations:** 1 Department of Animal and Genomics, Agricultural Biotechnology Research Institute of Iran (ABRII), North Region of Iran, Rasht, Iran; 2 Department of Medical Biotechnology, National Institute for Genetic Engineering and Biotechnology (NIGEB), Tehran, Iran; 3 Department of Animal and Poultry Science, College of Aboureyhan, University of Tehran, Tehran, Iran

**Keywords:** Multiplex PCR, Sex Determination, Free Fetal DNA, Maternal Plasma

## Abstract

**Background:**

In order to establish a reliable non-invasive method for sex determination in a bovine fetus in a routine setting, the possibility of identifying specific sequence in the fetal X and Y-chromosomes has been evaluated in maternal plasma using conventional multiplex polymerase chain reaction (PCR) analysis. The aim of this study was to provide a rapid and reliable method for sexing bovine fetuses.

**Materials and Methods:**

In this experimental study, peripheral blood samples were taken from 38 pregnant heifers with 8 to 38 weeks of gestation. DNA template was extracted by phenol-chloroform method from 350 µl maternal plasma. Two primer pairs for bovine amelogenin gene (bAML) and BC1.2 were used to amplify fragments from X and Y chromosomes. A multiplex PCR reaction has been optimized for amplification of 467 bp and 341 bp fragments from X and Y bAML gene and a 190 bp fragment from BC1.2 related to Y chromosome.

**Results:**

The 467 bp fragment was observed in all 38 samples. Both 341 and 190 bp fragments were detected only in 24 plasma samples from male calves. The sensitivity and specificity of test were 100% with no false negative or false positive results.

**Conclusion:**

The results showed that phenol-chloroform method is a simple and suitable method for isolation of fetal DNA in maternal plasma. The multiplex PCR method is an available non-invasive approach which is cost efficient and reliable for sexing bovine fetuses.

## Introduction

Invasive methods for prenatal diagnosis, include chorionic villus sampling (CVS) and amniocentesis that entail a risk of fetal loss and mortality. In 1997, Lo et al. (1) demonstrated the existence of fetal DNA in plasma and serum from healthy pregnant women. Recent studies have shown that fetal DNA in maternal plasma have a mean of 3.4% and 6.2% of total DNA in early and late gestation, respectively (2) and are cleared at an extremely rapid rate following birth (3). Fetal sex determination is now possible at 8 weeks of pregnancy, by testing maternal blood samples. The reasonable sensitivity in PCR techniques could be considered to detect small amounts of fetal DNA in maternal plasma, based on the identification of specific regions of X and Y chromosomes circulating in maternal blood. Recent technical advances enable us to use both intact fetal cells (4-7) and cell-free fetal DNA (8-10) in maternal plasma and serum for non-invasive fetal gender and also prenatal genetic identifications. However, amount of the fetal DNA that is obtained by these simple methods is not enough to reach the desired intention. Most of the technical improvements such as fluorescence-based polymerase chain reaction (PCR) (11) and real-time PCR (12-13) methods are highly sensitive and technically demanding. However, expensive equipment limits their application in a routine setting. Some conventional PCR analyses of maternal plasma, serum and blood using the Y-specific sequences for example; DYS14, (14) DYZ3 (15), DYZ1 (16), the Y-specific repeat sequences (17) and sex determination region Y (SRY) (18-20) have been introduced for the diagnosis of fetal sexing. But in a routine setting internal amplification control for examination of results is difficult to be interpreted (20). A synchronic amplification of the X-Y homologous region of the amelogenin in human (21) and bovine (22) is reported also, bovine zfx and zfy gene sequences in maternal blood using a pair primers have also been described for fetal sexing (23).

The aim of this study was to establish a rapid and reliable method for sexing of bovine fetuses.

This has prompted us to improve another non-invasive method of bovine fetal sex determination using multiplex PCR amplification of the X chromosome (467 bp amelogenin gene) and the Y chromosome (341 bp amelogenin gene fragments and BC1.2-sequence (that is derived from a male-specific bovine DNA sequences) simultaneously.

## Materials and Methods

### Blood sampling and plasma separation

In this experimental study, peripheral blood samples were taken from 38 pregnant heifers with gestational age of 8 to 38 weeks. Five normal heifers which had no history of pregnancy and five normal male cows served as positive control. Maternal peripheral blood sample (10 ml) were collected and put into tubes containing ethylenediaminetetraacetic acid (EDTA) (20 mM). The tubes were centrifuged at 1000 r/minute for 10 minutes with the brake and acceleration powers set to zero. Then tubes were centrifuged at 1200 r/minutes for 10 minutes with the brake and acceleration powers set to zero. Approximately 0.5 ml of supernatant (ie, the plasma) was left in the tube to ensure that the buffy coat was not disturbed. Tubes were centrifuged at 2000 r/minutes for 5 minutes with the brake and acceleration powers set to zero. 350 μl of supernatant and samples were stored at -20˚C for further processing.

### DNA extraction from plasma samples

Maternal plasma (350 μl) and an equal volume of Tris-EDTA (TE) buffer were mixed in a 1.5 ml Eppendorf tube by addition of 5 μl proteinase K solution (20 mg/ml). The mixture was digested at 56 ˚C for 3 hours, and then 350 μl of equilibrium phenol plus chloroform was added respectively. The tubes were centrifuged at 12000 r/minutes for 12 minutes and then the supernatant was transferred to a fresh tube. Equal volume of chloroform and isoamyl alcohol (24: 1) were added. After centrifugation (at 12000 r/minutes for 12 minutes), 1:10 of 3 mol/l sodium acetate and 2 volumes of 100% ethanol were added and the mixture was stored at -20˚C for 14 hours. Tubes were then centrifuged at 12000 r/minutes for 8 minutes at room temperature. The supernatant was discarded, DNA was purified and deposited with 70% ethanol before being dried in the airing closet. Tubes was dried at 65˚C for 3 minutes and were finally dissolved in 20 μl TE. Tube was placed in dry bath at 65˚C for 40 minutes and then stored at 4˚C.

### Concentration and purity of the extracted DNA

The concentration and purity of extracted DNA were identified by an ultraviolet spectrophotometer (Nanodrop 2000 Thermo). The results were read at 260 nm and 280 nm respectively.

### Amelogenin gene and BC1.2-sequnce amplification by multiplex PCR

In this study, two primer pairs were used: one set was derived from a male specific bovine DNA sequence termed, " BC1.2" (24). This primer amplified sex-determination Y chromosome and was thus representative of fetal DNA. The oligonucleotide sequence of the primers were: 5 ׳-ATCAGTGCAGGGACCGAGATG-3׳ and 5׳-AAGCAGCCGATAAACACTCCTT-3׳. This primer pair was designed to produce a 190 bp DNA fragment. The second primer pairs amplified of the bovine amelogenin (bAML) gene (22) on the X- and Y-chromosomes of bovine.The oligonucleotide sequences of the primers were: 5׳-AAATTCTCTCACAGTCCAAG-3׳ and 5׳-CAACAGGTAATTTTCCTTTAG-3׳. This primer was used to amplify a single fragment of 467-bp from the X-chromosome of female cattle and two fragments of 467-bp and 341-bp from the X- and Y-chromosomes of male cattle. The multiplex PCR reaction mixture (25 μl) contains 2.5 μl plasma DNA, 10 pmol of each primer (BC1.2 and bAML), 0.2 mM dNTPs, 1.5 mM MgCl2 and 5 U/μl Taq DNA polymerase (Roche), was added to each sample in a 0.2 ml tube. The DNA sequence was amplified by an initial denaturation step at 94 ˚C for 5 minutes, followed by 35 cycles of denaturation at 94 ˚C for 45 seconds, annealing at 54 ˚C for 60 seconds and extension at 72 ˚C for 60 seconds. The final extension was at 72 ˚C for 5 minutes. The 8 μl of PCR product and 2 μl loading buffer were mixed. The amplification products were analyzed by electrophoresis in 1.5% agarose gel and stained with ethidium bromide.

## Results

### Results of PCR

**Fig 1:**
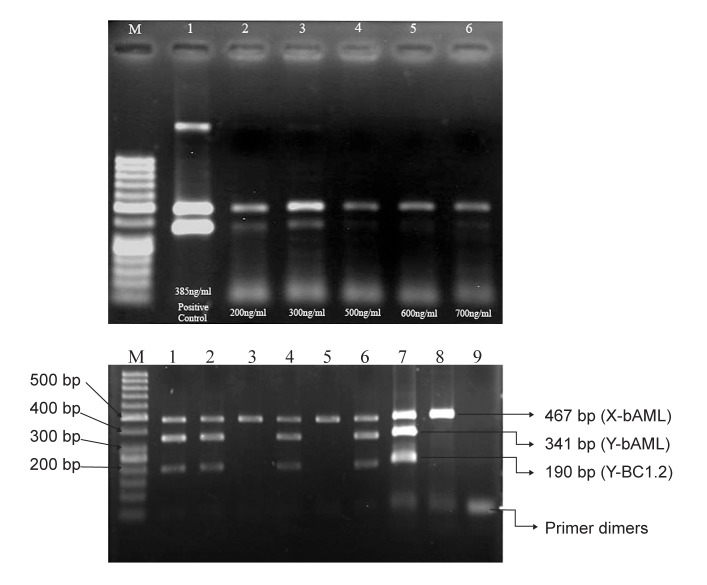
A. The concentration of template DNA (plasma DNA) extract of maternal plasma for PCR, respectivel Lane 2, 200 ng/ml; Lane 3, 300 ng/ml; Lane 4, 500 ng/ml; Lane 5, 600 ng/ml; Lane 6, 700 ng/ml; Lanes 1 is a adult male (positive control) genomic DNA and Lane M in figure A represents the 50 base pair ladder. B. Gel electrophoresis of bovine fetal sex prediction by a simultaneous multiplex PCR analysis of maternal plasma. The multiplex amplified products of the bAML sequence on X chromosome, the bAML sequence on Y chromosome and the BC1.2 sequence on Y chromosome are 467 bp, 341 bp and 190 bp in length, respectively. Result of multiplex PCR analysis on plasma DNA samples. Lanes 1-6 demonstrate the results of plasma DNA analysis of the 6 pregnant heifers. Lane 7 is made with adult male, and. Lane 8 is a normal heifer who had no history of pregnancy which served as positive control. Predictions of male pregnancies were made for 1, 2, 4 and 6 and female pregnancies for 3 and 5 respectively. Lane M in figure A represents the 50 base pair ladder.

The template DNA (plasma DNA) was diluted at different densities. When the concentration of template DNA extract of maternal plasma for mixture PCR was 200-300 ng/ml, there was the clearest band (Fig 1 A).

The results in figure 1B show three clear bands at 190 bp, 341 bp and 467 bp in the template DNA extracted from heifers bearing male fetus (lane 1,^2^,4 and 6). There is one band at 467 bp after the template DNA extracted from heifers that bearing female fetus amplified at the same condition (lane 3 and 5). After the extracting DNA samples from normal male cow and heifer who had no history of pregnancy respectively were amplified, positive (lane7 and 8) results were obtained. The results suggested that fetal DNA could be detected in heifers bearing a male fetus.

### Detectable rate of plasma 341 bp sequence bAML gene and BC1.2 sequence by multiplex PCR in maternal plasma samples

The DNA template was extracted from the plasma of 38 pregnant heifers (8 to 38 weeks). Fetus-derived Y sequence bAML gene and BC1.2 fragment were detected in 24 cases of maternal plasma samples from the 38 cases. The sensitivity of PCR product in 38 pregnant herifers was 100%.

## Discussion

The discovery of cell-free fetal DNA in maternal plasma in 1997 has opened up new possibilities for non-invasive diagnoses (1). Many related studies in humans (25-26), monkeys (27) and bovine (28) have proved that this fetal DNA is produced from the mechanism of cell transfer in the conceptus. However, there were reports of successful prediction of fetal sex through amplification of the male specific sequences (SRY) from the blood of pregnant cows (28). Thus, we have studied amelogenin gene and BC1.2 male specific bovine DNA sequence amplification in the maternal blood of pregnant cows.

In this study, we developed a multiplex PCR system for prenatal identification of fetal sexing. Though PCR methods cannot be compared with real-time PCR approach (12, 13), conventional PCR techniques provide a more practical methodology with acceptable sensitivity and specificity. The simple and simultaneous amplification of amelogenin gene on the X and Y chromosomes and the BC1.2 male specific bovine DNA sequence on the Y chromosome in a multiplex PCR system, respectively, could provide a satisfactory result for prenatal fetal determination. Moreover, it would be possible to accurately identify the fetal gender using PCR analysis of 38 maternal plasma samples during 8-38 weeks of gestation (Fig1 B and Table 1). No false-positive or false-negatives were generated at all trimesters of pregnancy among 38 pregnant heifers. In all pregnant heifers, the final accuracy of %100 was observed.

**Table 1:** Results of fetal sex prediction by non-invasive approach using the conventional multiplex PCR analysis of maternal plasma DNA in 38 pregnant heifers at various gestational ages


Samplesno	Gestational age(weeks)	Result ofmultiplex PCR	Birthoutcome	Samplesno	Gestational age(weeks)	Result ofmultiplex PCR	Birthoutcome

**1**	38.8	female	female	20	12.7	male	male
**2**	38.3	male	male	21	11.9	female	female
**3**	38.1	male	male	22	11.9	male	male
**4**	36.0	female	female	23	11.6	female	female
**5**	35.4	female	female	24	11.1	male	male
**6**	31.3	male	male	25	10.8	male	male
**7**	30.6	male	male	26	10.8	female	female
**8**	30.6	male	male	27	10.2	female	female
**9**	28.3	female	female	28	10.1	male	male
**10**	27.0	male	male	29	9.9	male	male
**11**	25.3	male	male	30	9.9	male	male
**12**	22.4	male	male	31	9.3	female	female
**13**	21.6	female	female	32	9.0	male	male
**14**	20.3	male	male	33	8.9	male	male
**15**	20.3	female	female	34	8.9	male	male
**16**	16.6	male	male	35	8.9	female	female
**17**	15.8	male	male	36	8.7	male	male
**18**	15.3	male	male	37	8.2	male	male
**19**	13.7	female	female	38	8.2	female	female


Unlike other described conventional PCR systems for prenatal fetal sex determinations on maternal plasma (15-16), the incorporation of the X-specific amelogenin gene amplification as an internal control of the multiplex PCR system herein described could greatly improve the reliability of the fetal sex identification. The use of multiplex PCR approach described here would be more practical in any laboratory where a conventional PCR is available. The ease, speediness and efficiency shown by this multiplex approach which requires no further modification of routine PCR procedure or additional advanced equipment should be directly applicable to a non-invasive prenatal fetal sex prediction by maternal plasma.

Like the results of similar studies in human (13, 19-20) and bovine (28-29), our results demonstrated that our fetal sex determination method using plasma is practical.

One of the benefits of this study and similar studies (16) was DNA extraction using phenol-chloroform method that costs lower than using an extraction kit. In addition, DNA that is extracted using phenol-chloroform method has appropriate quality and quantity.

## Conclusion

The results have shown that phenol-chloroform method is a simple and suitable method for isolation of fetal DNA in maternal blood. Furthermore, multiplex PCR technique is cost-efficient, reliable and available for non-invasive sex determination in bovine fetus.
